# From slit diaphragm to autoantigen formation: a SUMOylation-based perspective on minimal change disease

**DOI:** 10.3389/fneph.2025.1653595

**Published:** 2025-09-18

**Authors:** Emre Leventoğlu, Bahar Büyükkaragöz, Sevcan A. Bakkaloğlu

**Affiliations:** ^1^ Department of Pediatric Nephrology, Konya City Hospital, Konya, Türkiye; ^2^ Department of Pediatric Nephrology, Faculty of Medicine, Gazi University, Ankara, Türkiye

**Keywords:** minimal change disease, nephrin, post-translational modification, SUMOylation, anti-nephrin antibody-positive

Minimal change disease (MCD) remains one of the most common causes of nephrotic syndrome in children, characterized by dramatic steroid responsiveness and the absence of significant pathological findings on light microscopy. However, it is important to note that while nephrin is uniformly distributed along the glomerular basement membrane in a healthy kidney, biopsies from patients with MCD exhibit a granular staining pattern on electron microscopy. It also reveals a reduction in nephrin density within the foot processes, accompanied by its redistribution towards the cytoplasm. Additionally, MCD has been considered a disease with minimal or no immune involvement, particularly due to the lack of a massive immunoglobulin or complement deposition in kidney biopsy specimens (1). However, recent discoveries have begun to challenge this long-standing notion, particularly with the identification of anti-nephrin antibodies as potential contributors to the pathogenesis of the disease. In a study conducted by Watts et al. in 2022, nephrin autoantibodies were detected in the circulation during active disease and a significant decrease or disappearance of these antibodies was observed during treatment response. Furthermore, these autoantibodies were found to be associated with punctate IgG bound to podocytes in kidney biopsies (2). In a study conducted by Chen Q. et al. on patients diagnosed with MCD, anti-nephrin antibodies were detected in more than 25% of patients with nephrotic-level proteinuria, and it was shown that these antibodies became negative after treatment (3). Furthermore, anti-nephrin antibody positivity exceeds 75% in patients with steroid-dependent or frequent relapsing nephrotic syndrome. Rituximab treatment, which prevents antibody formation by destroying the B cell population, also achieves complete remission in the vast majority of patients (4). In a study conducted by Batal I. et al., it was reported that diffuse podocytopathy developed after transplantation in patients who were anti-nephrin antibody positive prior to kidney transplantation (5). These studies suggest that the development of autoantibodies against nephrin causes podocyte injury, leading to nephrotic-level proteinuria.

The development of auto-reactive antibodies against structural self-proteins, a hallmark of many autoimmune diseases, is a multifactorial process in which aberrant post-translational modifications (PTMs) play a pivotal role. By altering a protein’s structure after translation, PTMs can unmask cryptic epitopes or create neo-epitopes that are not normally visible to the immune system, rendering the modified proteins immunogenic. This mechanism, known as “autoantigenesis,” may lead to a breakdown of self-tolerance and the activation of autoreactive B and T cells. Although many PTMs are essential for normal protein function and cellular communication, certain aberrant modifications can disrupt antigen processing and presentation, thus facilitating the generation of pathological immune responses. Notably, it is estimated that between 50% and 90% of human proteins undergo PTMs, highlighting the widespread potential for such alterations to influence immune recognition (6).

The function of nephrin, an essential structural protein of the slit diaphragm in the glomerular filtration barrier, is tightly regulated by multiple PTMs, including phosphorylation, glycosylation, ubiquitination, proteolytic cleavage and SUMOylation. SUMOylation refers to the covalent attachment of Small Ubiquitin-like Modifier (SUMO) proteins to specific lysine residues on target proteins (7). A pivotal study demonstrated that nephrin is a substrate for SUMO modification both *in vitro* and *in vivo*. SUMOylation was shown to increase the steady-state level of nephrin and promote its expression at the plasma membrane. Notably, mutations of lysine residues at positions 1114 and 1224 in the intracellular domain of murine nephrin resulted in decreased protein stability and reduced surface expression. Furthermore, exposure of podocytes to ginkgolic acid, a pharmacological inhibitor of SUMOylation, led to diminished nephrin expression on the plasma membrane. Mice treated with ginkgolic acid exhibited marked proteinuria resembling that seen in MCD, underscoring the functional importance of nephrin SUMOylation in maintaining the glomerular filtration barrier integrity. Given that SUMOylation is reversible and dynamically regulated by cellular stress signals, it represents a compelling mechanism through which environmental or inflammatory stimuli might contribute to the pathogenesis of MCD (7).

A well-established example of autoantibody-induced disruption of a cell-cell adhesion complex is seen in the blistering skin disorder pemphigus. In this condition, pathogenic autoantibodies target desmogleins—key structural components of the desmosomal junctions, functionally comparable to nephrin in the slit diaphragm—leading to their internalization and removal from the cell surface. This results in desmosomal disassembly and a breakdown of intercellular cohesion (8). Interestingly, according to a PhD thesis in 2019, desmoglein-1 has to date been identified exclusively in multi-SUMO binding studies, highlighting a potential, though as yet unexplored, link between SUMOylation and desmosomal proteins (9). Indeed, a study in 2021 has demonstrated that desmogleins undergo SUMOylation (10). Therefore, we speculate that SUMOylation may regulate desmoglein stability, subcellular localization, and protein-protein interactions, all of which are essential for maintaining structural integrity in epithelial barriers. In both conditions, impaired SUMOylation may lead to destabilization of adhesion complexes—desmosomes in pemphigus and the slit diaphragm in MCD—resulting in loss of barrier function and clinical manifestations such as blistering or proteinuria (7, 8). This shared molecular vulnerability highlights SUMOylation as a potential unifying mechanism underlying structurally distinct yet functionally analogous autoimmune pathologies.

Taken together, these insights support a paradigm shift in our understanding of MCD, moving from a purely idiopathic glomerular disorder to one with identifiable autoimmune and molecular underpinnings. The identification of anti-nephrin antibodies and the central role of SUMOylation in maintaining nephrin stability highlight key mechanisms that may drive disease onset and progression. Elucidating these mechanisms will not only deepen our understanding of disease biology but also open new avenues for targeted therapeutic interventions.
